# Galvanostatic Entrapment of Penicillinase into Polytyramine Films and its Utilization for the Potentiometric Determination of Penicillin

**DOI:** 10.3390/s100402851

**Published:** 2010-03-29

**Authors:** Fatma Ismail, Samuel B. Adeloju

**Affiliations:** NanoScience and Sensor Technology Research Group, School of Applied Sciences and Engineering, Monash University, Churchill, Vic 3842, Australia; E-Mail: fatma.ismail@sci.monash.edu.au

**Keywords:** penicillin, penicillinase, polytyramine, biosensor, potentiometry

## Abstract

A sensitive and reliable potentiometric biosensor for determination of penicillin has been developed by exploiting the self-limiting growth of the non-conducting polymer, polytyramine. Optimum polytyramine-penicillinase (PTy-PNCnase) films for potentiometric detection of penicillin were accomplished with monomer solutions which contained 0.03 M tyramine, 37 U/mL penicillinase, 0.01 M KNO_3_, and 3 mM penicillin with an applied current density of 0.8 mA/cm^2^ and an electropolymerisation time of 40 seconds. The potentiometric biosensor gave a linear concentration range of 3–283 μM for penicillin and achieved a minimum detectable concentration of 0.3 μM. The biosensor was successfully utilized for the detection of Amoxycillin and gave an average percentage recovery of 102 ± 6%. Satisfactory recoveries of penicillin G were also achieved in milk samples with the potentiometric biosensor when concentrations are ≥20 ppm.

## 1. Introduction

**Figure 1.**
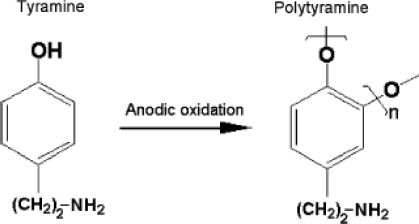
Polymerisation process of tyramine. Reproduced from [6].

**Figure 2.**
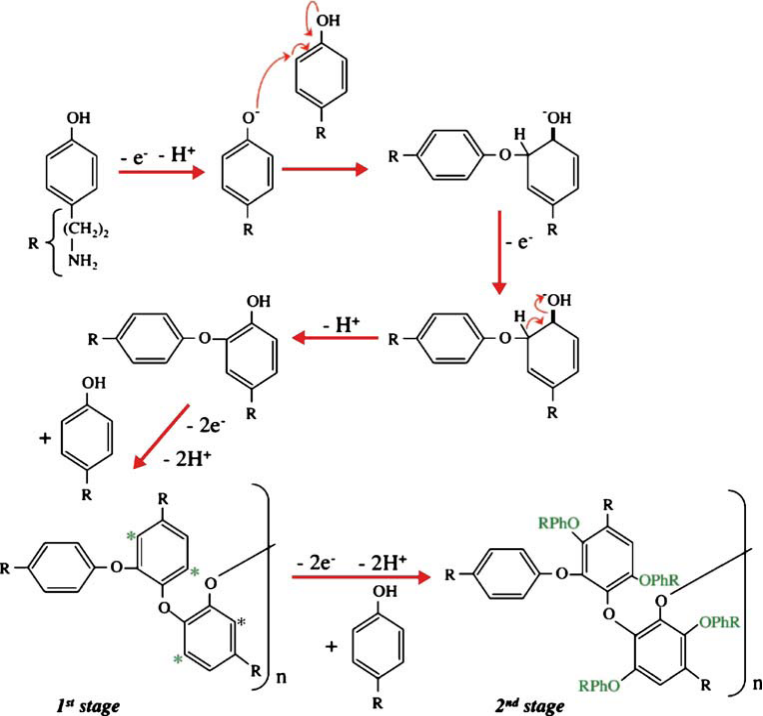
Electrode mechanism for polymerisation of tyramine. Reproduced from [7].

Tyramine or 4-(2-aminoethyl) phenol is a biogenic amine derivative of tyrosine which occurs naturally in many foods especially in fermented food. It is not hazardous unless ingested in large amounts or in cases where normal catabolism processes are inhibited [1]. Tyramine has also been identified as a versatile monomer for formation of non-conducting polymer and fabrication of biosensors [2–11]. The process and mechanism for electropolymerisation of tyramine, which occurs in the ortho-position to the activating hydroxyl group, are shown in Figures 1 and 2 [2–4]. The presence of the free amine groups on the polymer backbone enables covalent attachment of enzymes through the formation of a peptide bond [5].

Nevertheless, very few studies have been reported to date on the use of polytyramine (PTy) for the entrapment of enzymes and fabrication of biosensors [3,5,6,8–11]. One of the methods used for the entrapment of enzymes involves electropolymerisation of tyramine, as illustrated by a detailed mechanism in Figure 2. The first step of this process involves the formation of a radical-cation and dimerisation process involving 2 electrons and the loss of 2 protons. The linear chain polymerisation produces relatively short oligomers with little or no conductivity. This is followed by an increase in the size of the oligomer which is characterised by the formation of a smooth film. Oligomeric chains can be linked through the reactive sites which are denoted by stars, as shown in Figure 2. The polymerisation process proceeds through these sites and the oxidation of the monomer continues to produce a polytyramine film.

Polytyramine films have also been used in drug release matrices, where enzymes or oligonucleotides have been cross-linked, entrapped or covalently attached. This is facilitated by the availability of the primary amine group, as well as by the mild polymerisation conditions [3,12–15]. In cases where non-conducting polymers, such as polytyramine, have been employed as alternative matrices for fabrication of biosensors, they have produced sensitive biosensors with a rapid response time due to the self-limiting growth of the resulting non-conducting polymers. This behaviour has been shown to promote formation of a thinner film with a more efficient diffusion rate [16,17]. Non-conducting polymers also have the added advantage of being permselective and able to prevent interferants from fouling the electrode surface. Furthermore, the thickness of a non-conducting layer is usually 10–100 nm thick and, as such, often enabled rapid diffusion of the substrate to and from the membrane [15]. This also influences the achievable linear concentration range and sensitivity of the biosensor, depending on other factors, such as the enzyme concentration, pH and buffer concentration.

Some of the methods reported for the fabrication of polytyramine biosensors include the modification of a gold electrode by electrochemical polymerisation into the defect sites of a hexadecanethiol monolayer [6]. Other methods have included the covalent binding of the enzyme via carbodiimide coupling after electropolymerisation [5]. The covalent attachment of glucose oxidase to the free amine groups on the polytyramine film [3], as well as the attachment of sulphite oxidase, lactate oxidase and L-amino acid oxidase to the polymer chain, are amongst some of the reported methods [5]. Also enzymes such as L-amino acid oxidase [9], tyrosinase [10], and lactate oxidase [11] have been cross-linked to polytyramine with glutaraldehyde. In another case, a glucose biosensor was fabricated through the modification of a glassy carbon (GCE) and carbon screen printed (SPE) electrodes with rhodium, GOx and polytyramine [18]. Potentiodynamic deposition of polytyramine has also been achieved on a graphite substrate, followed by the deposition of platinum as a means of obtaining a platinum-polytyramine composite [19].

In this study, we explore, for the first time, the use of polytyramine for the fabrication of a potentiometric penicillin biosensor. The entrapment of penicillinase into a polytyramine matrix will be attempted through galvanostatic polymerisation of tyramine in the presence of the enzyme. The optimization of the resulting PTy-PNCnase films for potentiometric detection of penicillin will involve consideration of the effects of various parameters, such as the influence of the supporting electrolyte, penicillinase concentration, tyramine concentration, applied current density, polymerisation time and buffer concentration. The ability to use the potentiometric biosensor for reliable determination of penicillin in a pharmaceutical medication (Amoxycillin) and in milk samples will also be investigated.

## 2. Materials and Methods

### 2.1. Reagents and Chemicals

Penicillinase (3.5.2.6) from Bacillus Cereus was purchased from Sigma Aldrich (USA) and was used to prepare a stock solution (74 U/mL) by dissolving in Milli-Q water. Penicillin G was purchased from Sigma Aldrich (USA) and a stock solution of 0.01 M penicillin G was prepared and stored in the refrigerator.

A phosphate buffer solution was prepared by neutralising 0.05 M phosphoric acid with sodium hydroxide to pH 8. 0.1 M KNO_3_ was added to the buffer as the supporting electrolyte and the pH of the buffer was adjusted as required. The buffer solution was stored in the refrigerator and diluted further as required.

Tyramine (4-hydroxyphenethylamine) was purchased from Sigma Aldrich and 1.3272 g was dissolved in Milli-Q water, with 1 mL orthophosphoric acid (85%) added and made up to 100 mL to give 0.1 M solution.

### 2.2. Instrumentation/Electrodes

A potentiostat/galvanostat was employed for electropolymerisation and potentiometric measurements. Platinum electrodes were polished prior to use with 0.3 μm alumina on a micropolishing cloth and then rinsed with Milli-Q water. The electropolymerisation of monomer solutions was performed galvanostatically in a three-electrode cell which consisted of a platinum working electrode (0.08 cm^2^), platinum auxiliary and reference electrodes. Under optimum conditions, the monomer solution contained 0.03 M tyramine, 37 U/mL of penicillinase, 3 mM penicillin and 0.01 M KNO_3_. A current density of 0.8 mA/cm^2^ was applied for 40 s.

### 2.3. Potentiometric Biosensing

A two-electrode electrochemical cell was used for all potentiometric measurements. Solutions were stirred during potentiometric measurements with a Sybron Thermolyne stirrer (model S-17410). Potentiometric measurements at each concentration were repeated 3 times.

### 2.4. Preparation of Milk Samples

Homogenized and pasteurised milk (total fat content 3.4 %, saturated fat content 2.2 %) was purchased from a supermarket (Sydney, NSW). Four milk samples (30 mL each) were spiked with 0, 250, 500 and 1000 μL of 0.01 M penicillin G, respectively. Acid fractionation was then applied to each sample by adding 0.1 M hydrochloric acid until the pH reached 4.6. This enabled the proteins to be separated into 2 components, namely; caseins in the precipitate and whey proteins in the supernatant which was extracted using a pasteur pipette leaving behind an insoluble precipitate, the caseins. The supernatant was then centrifuged at 6000 rpm for 25 minutes and filtered through 0.1 μM Whatman filter paper no. 541. A clear solution which resulted was diluted with 15 mL of 0.01 mM phosphate buffer. This procedure was repeated for all 4 samples and milk samples were refrigerated at 4 °C.

### 2.5. Preparation of Amoxycillin Samples

Twelve Amoxycillin 500 mg tablets were ground into a fine powder. The powdered samples were then transferred to a 100 mL beaker and dissolved in 0.01 mM phosphate buffer solution. The sample was then filtered using a 0.1 μM 541 Whatman filter paper and the filtrate was collected and centrifuged at 2000 rpm for 10 minutes.

The concentration of Amoxycillin was determined by injecting a 30 μL aliquot of the dissolved sample into the buffer solution. This was followed by sequential additions of 100, 200 and 800 μL of 0.01 M penicillin G standard solution.

### 2.6. Preparation of Samples for XPS Analysis

The polytyramine film was prepared as indicated in section 2.3, but to obtain sufficiently thick films, a polymerization time of 300 s and an applied current density of 0.9 mA/cm^2^ were used. XPS analysis was performed by using a KRATOS Analytical AXIS-HSi at the CSIRO Clayton Laboratories, Melbourne, Australia. Survey spectra were performed to determine the elements present on the surface of the films. The analysis was conducted with a monochromatic rotating anode source which provided A1Kα radiation (12 kV × 15 mA). The pressure in the analysis chamber was 2 × 10^−8^−5 × 10^−8^ mbar. The angle between the normal to the sample surface was 0°. The area of analysis of the film was ca. 1 millimetre and probed at a sampling depth of ca.10 nanometres.

### 2.7. Preparation of Samples for SEM Analysis

Polytyramine films were electropolymerised on aluminium coated electrodes (4 cm × 1 cm). The monomer solution contained 0.03 M tyramine, 37 U/mL penicillinase, 0.003 M penicillin and 0.01 M KNO_3_. Other polymerization conditions were as given above in section 2.6. SEM analysis was carried out using a JEOL, JSM-6300F scanning electron microscope. The morphologies of the films were characterised at an acceleration voltage of 15 kV and magnifications of 5, 000X, 10, 000X and 20, 000X.

### 2.8. Titration Procedure

For comparison, a titrimetric method was used as described in this section. 500 mg Amoxycillin tablets were crushed and 1.08 g was transferred to a 100 mL volumetric flask, dissolved and diluted to mark with 0.01 mM phosphate buffer. The mixture was then centrifuged for 10 minutes at 6, 000 rpm. 10 mL of this solution was transferred to a 250 mL volumetric flask and 40 mL of concentrated HCl and 10 mL chloroform were added. The mixture was then titrated with 0.05 M potassium iodate with vigorous shaking. The colour of the chloroform layer changed from colourless to brown/deep red to colourless again. The end point was taken as the first permanent decolourisation of the chloroform layer. This procedure was repeated for three tablets.

## 3. Results and Discussion

### 3.1. Surface Characterisation of PTy-PNCnase Films by SEM and XPS

**Figure 3.**
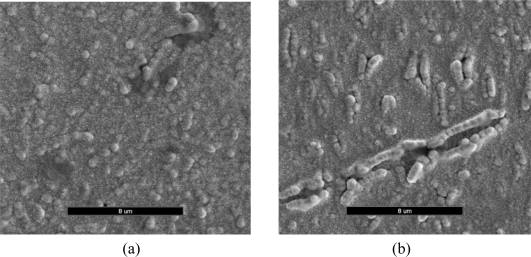
Scanning electron micrographs obtained for PTy films in the (a) absence and (b) presence of PNCnase (37 U/mL). Monomer solution contained 0.03 M tyramine, 0.003 M penicillin and 0.01 M KNO_3_. Electropolymerization conditions were: current density of 0.9 mA/cm^2^ and polymerization time of 5 minutes. Magnification was x5,000 in both cases.

The morphological changes caused by the incorporation of penicillinase into the PTy film was investigated by SEM. Figure 3 shows that the scanning electron micrographs obtained for the PTy films in the absence and presence of PNCnase. The surface morphology obtained for PTy film in the absence of penicillinase was more uniform (Figure3a), whereas that obtained in the presence of the enzyme was distinctly different showing several nodular protrusions that are rod-like and clumped together (Figure3b). The presence of these larger nodules suggests that PNCnase was incorporated in the polytyramine film.

Further confirmation of the presence of PNCnase in the PTy film was investigated by XPS analysis. The key elements and associated peak assignments identified from the spectra are summarized in Table 1. All spectra were referenced by setting the hydrocarbon C 1s peak to 285.0 eV to compensate for the residual charging effects [31]. The presence of penicillinase in the film was characterized by the observed S 2p peak at 168 eV [32]. This sulfur peak is related to the amino acids present in the enzyme and was not observed in PTy films prepared in the absence of PNCnase. However, in both cases, the C 1s (1,2) peak observed at 285 eV corresponds to the C-C bond of the reference carbon. The presence of N 1s peak at 398.3 eV is indicative of the N-H bond present in polytyramine [2]. The C 1s (3) peak at 286.5 eV is also indicative of the C-O bonds present in polytyramine. The C 1s (4) peak at 288.3 eV is due to N-C=O or C=O bonds associated with the anodic oxidation of tyramine [6].

The results of the XPS analysis, used in conjuction with the morphological changes observed by SEM, confirmed the presence of the enzyme in the PTy-PNCnase film. However, the use of this film for optimum potentiometric detection of penicillin still requires optimization of: (a) the galvanostatic polymerisation conditions for film formation, and (b) the measurement conditions for potentiometric detection. The influence of each of the important parameters in both the film formation and measurement conditions on the potentiometric detection of penicillin was therefore carefully investigated. The results of the optimum conditions are discussed below separately for film formation and measurement conditions.

### 3.2. Optimization of Galvanostatic Polymerization Conditions

#### 3.2.1. Influence of Tyramine and Penicillinase Concentrations

**Figure 4.**
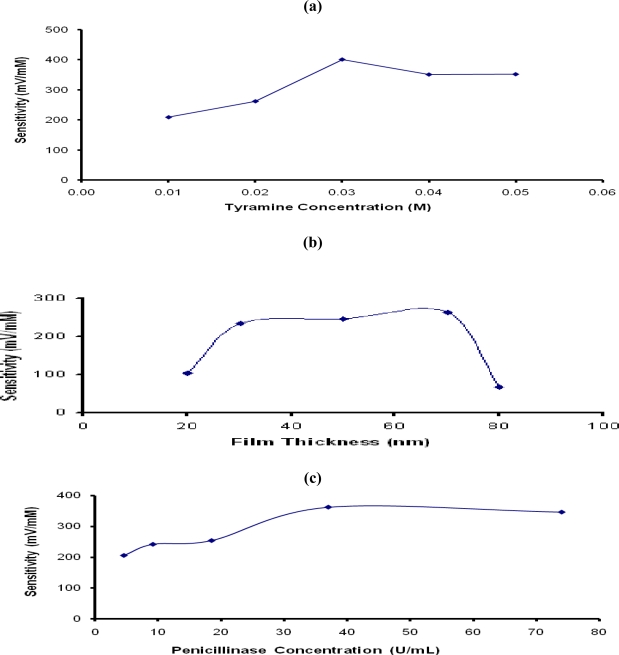
Influence of (a) tyramine concentration, (b) film thickness, and (c) penicillinase concentration used for formation of PTy-PNCnase film on penicillin response. Film formation conditions: (a) 19 U/mL penicillinase, 0.01 M penicillin, applied current density 0.9 mA/cm^2^, electropolymerization time 40 seconds, and tyramine concentration was varied; (b) same as (a), except that 0.03 M tyramine was added and penicillinase concentration was varied.

The influence of tyramine (Ty) concentration used for formation of the PTy-PNCnase film on the sensitivity of penicillin potentiometric response is shown in Figure 4a. Evidently, the response of the sensor increased progressively with Ty concentration up to 0.03 M and adequate surface coverage of the electrode was observed at this monomer concentration. The lower sensitivities observed at the lower Ty concentration (<0.03 M) was due to the combination of incomplete surface coverage and incorporation of lower PNCnase in the film. Beyond 0.03 M Ty, the sensitivity of penicillin potentiometric response declined slightly due to increased film thickness which also increased the diffusion barrier. Figure 4b shows that the sensitivity of the penicillin response increased with increasing film thickness up to an optimum value at 70 nm, but decreased rapidly at a thickness of 80 nm. The resulting denser films were less permeable and restricted the amount of the catalytic product detected by the electrode [20]. It was therefore concluded that the optimum penicillin potentiometric response was obtained with 0.03 M Ty and this monomer concentration was used for all other investigations.

The sensitivity of the penicillin potentiometric response obtained with the PTy-PNCnase film will also be influenced by the entrapped enzyme concentration [5,21]. Figure 4c shows that the potentiometric response increased gradually with increasing PNCnase concentration in the monomer solution up to 37 U/mL. At the lower enzyme concentrations in the monomer solution, the sensitivity of the penicillin potentiometric response was affected by the lower PNCnase present in the PTy-PNCnase film, as well as the film thickness. At such low catalytic activity, the biosensor is kinetically limited and the enzymatic reaction will tend to occur within the polymer layer [5]. Interestingly, the addition of PNCnase concentrations >37 U/mL into the monomer solution did not result in further increase in sensitivity of the penicillin potentiometric response. This is due partly to the increase in film thickness which increased the diffusion barrier, limiting the ability of the catalytic product to reach the electrode and, hence, decreasing the sensitivity of the electrode [5]. This may also be due to the saturation of the PTy-PNCnase film with the enzyme. For these reasons, all PTy-PNCnase films used in other investigations were produced with monomer solutions which contained 37 U/mL of penicillinase.

#### 3.2.2. Effect of Addition of a Supporting Electrolyte and Penicillin

**Figure 5.**
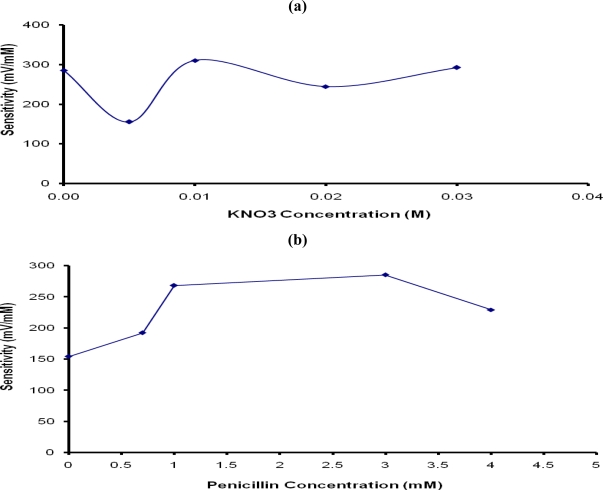
Effect of (a) KNO_3_ and (b) penicillin concentrations used for the formation of PTy-PNCnase film on the sensitivity of penicillin potentiometric response. Film formation conditions were as for Figure 4, except that for: (a) 0.03 M tyramine and 37 U/mL penicillinase were used while KNO_3_ concentration was varied; (b) same as (a), but 0.01 M KNO_3_ was added and penicillin concentration was varied.

Supporting electrolytes, such as potassium chloride and potassium nitrate are often added to monomer solution to increase electrical conductivity and improve the sensitivity of biosensors [20,22,23]. In this study, potassium nitrate (KNO_3_) was used to make the PTy-PNCnase film conductive. The results in Figure 5a show that the addition of 0.005 M KNO_3_ to the monomer solution lowered the penicillin potentiometric response, possibly due to the transition from a non-conductive to a mildly conductive film. However, with further addition of KNO_3_ and associated increase in the film conductivity, the sensitivity of the penicillin potentiometric response increased again, reaching an optimum with the addition of 0.01 M to the monomer. Beyond this KNO_3_ concentration, the sensitivity of the penicillin potentiometric response decreased slightly due to increased film thickness caused by the increased film conductivity and diffusion barrier. For this reason, 0.01 M KNO_3_ was added to the monomer solution to make the PTy-PNCnase film conductive.

Unfortunately, the potentiometric response obtained for penicillin with the PTy-PNCnase electrode was unstable and decreased rapidly after reaching a maximum potential value with each penicillin addition. Although it was still obvious that the penicillin potentiometric response increased with increasing penicillin concentration, a steady state response was not achieved and the quality of the response was inadequate to ensure reliable quantification of penicillin. It was suggested in a previous study [26] that this decline in the potentiometric response occurs when the pH of the membrane is decreased due to the negative charge on the membrane. Such a decrease in the membrane pH may result from the retention of penicilloate produced from the catalytic reaction on the membrane. The resulting charge on the membrane has an electrostatic influence on the enzyme and the substrate [26], resulting in a decrease in the sensitivity of the electrode. It was expected that the addition of small amounts of penicillin into the monomer solution used for the formation of the PTy-PNCnase film may be beneficial in reducing this effect. The results in Figure 5b shows that addition of 0.5–3 mM penicillin resulted in a significant improvement in the sensitivity of penicillin response and enabled attainment of a steady state response. The optimum sensitivity of the penicillin potentiometric response was obtained with PTy-PNCnase film prepared with a monomer solution which contained 3 mM penicillin. It appears that the presence of penicillin in the film influences the charge on the membrane and, hence, increases the conductivity of the polytyramine membrane. The more positive charge induced by penicillin on the membrane improves its ability to attract the substrate, penicillin, which in turn enhances the sensitivity of the potentiometric biosensor. The addition of penicillin concentrations higher than 3 mM into the monomer resulted in a slight decline in sensitivity of penicillin response due to the saturation of the film with penicillin. Alternatively, the excess penicillin may react with some of the PNCnase and reduce the amount of enzyme available for the potentiometric detection. For this reason, the PTy-PNCnase film used for potentiometric detection of penicillin was prepared with monomer solution which contained 3 mM penicillin.

#### 3.2.3. Effect of the Applied Current Density and Polymerisation Time

**Figure 6.**
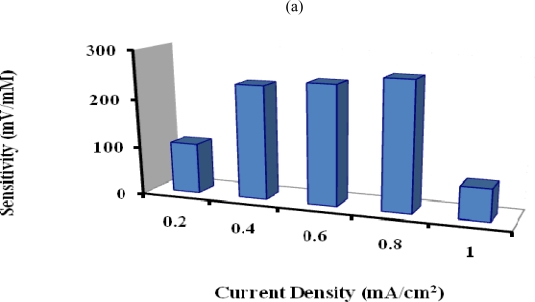

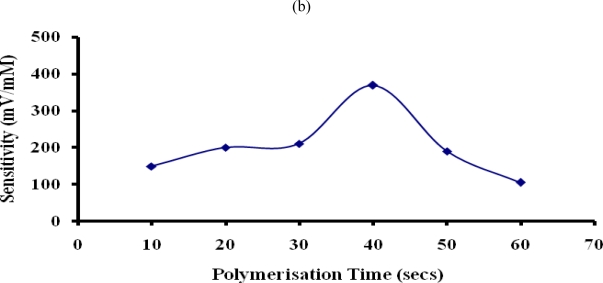
Effect of (a) applied current density and (b) polymerization time used for formation of PTy-PNCnase film on sensitivity of penicillin potentiometric response. Film formation conditions were as for Figure 5, except that for: (a) 3 mM penicillin and 0.01 M KNO_3_ were used, while the applied current density was varied; (b) same as (a) but an applied current of 0.8 mA/cm^2^ was used and the polymerization time was varied.

The penicillin potentiometric response obtained with the PTy-PNCnase electrode can also be influenced by the applied current density used for film formation and, hence, by the film thickness. Figure 6a shows that the sensitivity of the penicillin potentiometric response increased progressively with increasing applied current density up to an optimum at 0.8 mA/cm^2^. This is due to increasing PTy-PNCnase film thickness and associated increase in the amount of penicillinase entrapped in the polymer film. Beyond this applied current density, the sensitivity of the penicillin response was limited by further increase in film thickness. Consequently, a substantial decrease in sensitivity of the response occurred due to the increased diffusion barrier [28]. Also, the substantial drop in sensitivity observed when an applied current density of 1 mA/cm^2^ was employed for film formation suggests that the more rapid formation of the self-limiting polymer may have affected the rate of entrapment of penicillinase in the film. All PTy-PNCnase films used for all subsequent investigations were therefore formed with an applied current density of 0.8 mA/cm^2^.

The polymerisation time used for the film formation may also influence the amount of enzyme entrapped in the polymer film [28]. Figure 6b shows that the sensitivity of the penicillin potentiometric response increased substantially with the use of increasing polymerisation time for formation of PTy-PNCnase film up to 40 secs. This is again due to increasing film thickness and associated increase in the amount of PNCnase incorporated. However, beyond this polymerisation time, a significant decrease in the sensitivity of the penicillin response was observed due to increased film thickness and associated increase in the diffusion barrier which limits the ability of the catalytic product to reach the electrode surface [33]. A polymerisation time of 40 seconds was therefore used for formation of PTy-PNCnase films for optimum potentiometric detection of penicillin.

### 3.3. Optimization of Measurement Condition

**Figure 7.**
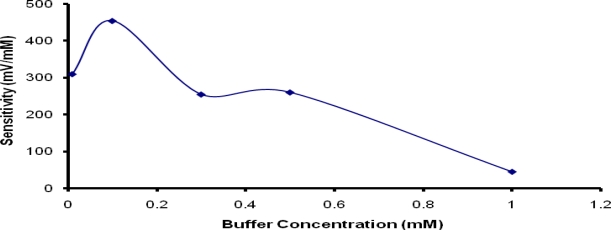
Influence of buffer concentration on sensitivity of penicillin potentiometric response. Film formation conditions were as for Figure 6.

Two main factors that can influence the sensitivity of potentiometric detection with biosensors are buffer concentration and pH of the measurement solution [29]. Due to the use of PNCnase for fabrication of the penicillin biosensor, the required pH was fixed at 7 to ensure optimum enzymatic activity [34]. However, the buffer concentration used for the potentiometric measurement can have significant effect on the detection of the relatively small concentration of H^+^ ion produced during the catalytic reaction. Figure 7 shows that the sensitivity of the potentiometric response increased with the addition of 0.1 mM buffer to the measurement solution, but diminished rapidly with increasing buffer concentration up to only 1 mM. This suggests that the concentration of hydrogen ions produced from the dissociation of penicilloic acid was <0.1 mM. The use of buffer concentrations greater than this level provide much higher buffering capacity which makes it difficult to detect the much lower H^+^ ion concentration produced. As indicated in a previous study [30], higher buffer concentrations increase the effective diffusion coefficient of hydrogen ions and shorten the response time. Both of these effects contributed to the reduction of the potentiometric response as observed in this study. This observation highlights the need to balance the achievement of sensitive potentiometric measurement against the necessity to control the buffering capacity of measurement solution [29].

### 3.4. Analytical Applications

**Figure 8.**
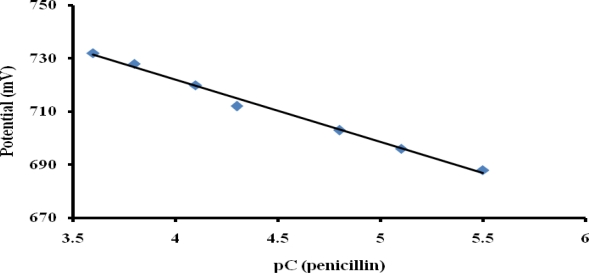
Plot of potential *versus* logarithmic concentration of penicillin obtained with the PTy-PNCnase biosensor. Film formation conditions were as for Figure 6 and measurement solution was 0.1 mM buffer.

To ensure optimum potentiometric detection of penicillin, all measurements were performed in 0.1 mM buffer solution and as little as 0.3 μM penicillin was detected in this medium with a response time of 21 seconds. Figure 8 shows that the penicillin potentiometric response obtained with the optimized PTy-PNCnase biosensor increased with increasing penicillin concentration from 3 to 283 μM. The line equation for the calibration curve is y = 23.463x + 815.86 and the R2 value of 0.9923 demonstrates a good linear relationship. The upper linear range can be extended by using higher buffer concentration, but this also increased the detectable concentration at the lower linear range [30].

**Figure 9.**
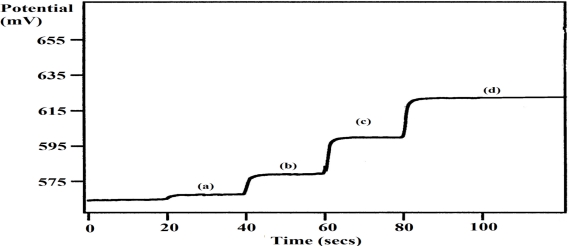
Potentiograms obtained for the analysis of Amoxycillin tablet with the PTy-PNCnase biosensor. Measurement solution was 0.01 mM buffer with the addition of: (a) 30 μL Amoxycillin sample solution, (b) +100 μL, (c) +200 μL, and (d) +800 μL penicillin standard.

The application of the PTy-PNCnase biosensor to the analysis of Amoxycillin tablets proved to be successful. Figure 9 shows that a potentiometric response was obtained with the injection of the Amoxycillin sample solution into the buffer solution and this response increased further with increasing addition of a penicillin standard solution. These observations provided both qualitative and quantitative evidences for the potentiometric detection of penicillin in the tablet with the biosensor. The results in Table 2 show that an average percentage recovery of 102 ± 6% was obtained with the biosensor, which was in close agreement with that obtained with the standard titrimetric method (105 ± 5%). The presence of clavulanic acid which has weak antibiotic properties and acts as a competitive inhibitor, despite being structurally similar to β-lactam antibiotics, may also influence the percentage recoveries obtained by both methods [34]. Also, although the reproducibility obtained for the analysis of Amoxycillin tablets were similar, the use of the PTy-PNCnase biosensor was less time consuming.

The data in Table 2 also shows that the titrimetric method gave an average percentage recovery of 105 ± 5% for the analysis of 500 mg Amoxycillin tablets. The presence of sodium starch glycollate in the tablets may have influenced the results to some extent [33], but this level of recovery is acceptable and does not necessitate its removal.

The PTy-PNCnase biosensor was also applied to the potentiometric determination of penicillin in four separate milk samples, prepared as described in the experimental section. However, the recovery of penicillin in the milk samples was far more complex and not reproducible, as demonstrated by the data in Table 3. This behaviour may be attributed to the complex composition of milk and the tendency of penicillins to bind to the hydrophobic sites of milk proteins [35]. The presence of milk proteins may also result in non-specific binding with whey proteins and cause interferences with the potentiometric measurements with the biosensor [36]. The improvement in the reproducibility of the recovery of penicillin in milk with higher spiked penicillin concentrations (>10 ppm) suggests that the effect of such interferences is reduced when higher penicillin concentrations are presented. The achievement of a 90 ± 14% recovery for a 20 ppm spike suggests that reasonable recovery of penicillin in milk could be achieved with the PTy-PNCnase biosensor when ≥20 ppm penicillin is present. However, further work is required on the sample preparation of milk samples to enable reliable determination of <20 ppm penicillin in milk samples with the PTy-PNCnase biosensor.

## 4. Conclusions

The galvanostatic entrapment of penicillinase into polytyramine film has been successfully demonstrated for fabrication of a PTy-PNCnase biosensor for reliable potentiometric detection of penicillin. Evidence of the presence of the enzyme was obtained by both SEM and XPS analysis of the PTy-PNCnase films. This was also further supported by the attainment of potentiometric responses with increasing penicillin concentrations. The minimum detectable concentration of 0.3 μM is far superior than 5–10 μM reported for other penicillin biosensors [38–41]. Although the lifetime of the sensor was not investigated, repeated use of the biosensor was achieved and changes in sensitivity were compensated for by using standard additions for quantification. Reliable determination of penicillin in Amoxycillin tablets was achieved with the biosensor, but further work is required to remove or reduce interferences in the determination of penicillin in milk samples.

## Figures and Tables

**Table 1.** Mean Binding Energy and Assignments of XPS Peaks Obtained for PTy-PNCnase Film.

**Element**	**Binding Energy** (eV)	**Standard Deviation** (eV)	**Peak Assignment**
C 1s (1,2)	285.0	0.0	hydrocarbons CH_x_ reference carbon
C 1s (3)	286.5	0.1	C-O, C-N
C 1s (4)	288.3	0.0	C=O
O 1s	531.5	0.1	C-O
N 1s	398.3	0.1	N-H
S 2p	168.0	0.8	Sulfur

**Table 2.** Comparison of the Recoveries of Penicillin in Amoxycillin Tablets by PTy-PNCnase Biosensor and Titrimetric Method.

**Amount in Tablet** (mg)**^a^**	**Conc. Found *Biosensor*** (mg)**^b^**	**% Recovery *Biosensor***	**Conc. Found *Titration*** (mg)**^c^**	**% Recovery *Titration***
Amoxycillin 500 Clavulanic Acid 125	544 ± 43	109 ± 9	525 ± 20	105 ± 4
Amoxycillin 500 Clavulanic Acid 125	466 ± 32	93 ± 6	545 ± 40	109 ± 8
Amoxycillin 500 Clavulanic Acid 125	526 ± 15	105 ± 3	505 ± 20	101 ± 4
**Average**	**512 ± 30**	**102 ± 6**	**525 ± 27**	**105 ± 5**

a Dosage specified by pharmaceutical company

b Mean of four values (n = 4) obtained with a 95% confidence limit

c Mean of three values (n = 3) obtained with a 95% confidence limit

**Table 3.** Recovery of Penicillin G in Milk Samples with PTy-PNCnase Biosensor.

**Penicillin G Added** (ppm) **^a^**	**Penicillin G Found** (ppm) **^b^**	**% Recovery**
1	1.1 ± 0.8	110 ± 80
5	4.6 ± 1.6	92 ± 32
10	7.8 ± 0.8	78 ± 16
20	18 ± 2.7	90 ± 14

a Spiked concentration of penicillin G in milk sample.

b Mean of four values (n = 4) obtained with biosensor with a 95% confidence limit.

## References

[1] Yigit M., Ersoy L. (2003). Determination of tyramine in cheese by LC-UV. J. Pharm. Biomed. Anal.

[2] Dubois J.E., Lacaze M.C., Pham M.C. (1981). Obtaining thin film of “reactive polymers” on metal surfaces by electrochemical polymerization. Part III. Amino substituted polyphenylene oxide films. Application to preparation of ferrocene electroactive films. J. Electroanal. Chem.

[3] Situmorang M., Gooding J.J., Hibbert D., Barnett D. (1998). Electrodeposited polytyramine as an immobilisation matrix for enzyme biosensors. Biosens. Bioelectronics.

[4] Cole M., Thissen H., Losic D., Voelcker N.H. (2007). A new approach to the immobilisation of poly (ethylene oxide) for the reduction of non-specific protein adsorption on conductive substrates. Surf. Sc.

[5] Situmorang M., Gooding J.J., Hibbert D.B., Barnett D. (1999). Immobilisation of enzyme throughout a polytyramine matrix: a versatile procedure for fabricating biosensors. Anal. Chim. Acta..

[6] Losic D., Cole M., Thissen H., Voelcker N.H. (2005). Ultrathin polytyramine films by electropolymerisation on highly doped p-type silicon electrodes. Surf. Sc.

[7] Tenreiro A.M., Nabais C., Correia J.P., Fernandez F.M.S.S., Romero J.R., Abrantes L.M. (2007). Progress in the understanding of tyramine electropolymerisation mechanism. J. Solid State Electrochem.

[8] Tsui I., Eguchi H., Yasukouchi K., Unoki M., Taniguchi I. (1990). Enzyme immunosensors based on electropolymerized polytyramine modified electrodes. Biosens. Bioelectronics.

[9] Cooper J.C., Schubert F. (1994). A biosensor for L-amino acids using polytyramine for enzyme immobilization. Electroanalysis.

[10] Debenedetto G.E., Palmisano F., Zambonin P.G. (1996). Flow-through tyrosinase enzyme reactor based on reticulated vitreous carbon functionalized by an electrochemically synthesized film. Anal. Chim. Acta.

[11] Palmisano F., De Benedetto G.E., Zambonin C.G. (1997). Lactate amperometric biosensor based on an electrosynthesized bilayer film with covalently immobilized enzyme. Analyst.

[12] Tenreiro A., Cordas C.M., Abrantes L.M. (2003). Oligonucleotide Immobilisation on Polytyramine-Modified Electrodes Suitable for Electrochemical DNA Biosensors. Portug. Electrochim. Acta.

[13] Tran L.D., Piro B., Pham T., Ledoan C., Angiari C., Dao Le. H., Teston F. (2003). A polytyramine film for covalent immobilization of oligonucleotides and hybridization. Synth. Met.

[14] Suprun E.V., Budnikov H.C., Evtugyn G.A., Brainina Kh. Z. (2004). Bienzyme sensor based on thick-film carbon electrode modified with electropolymerised tyramine. Bioelectrochemistry.

[15] Miao Y., Chen J., Hu Y. (2005). Electrodeposited non-conducting polytyramine for the development of glucose biosensors. Anal. Biochem.

[16] Nakabayashi Y., Wakuda M., Imai H. (1998). Amperometric Glucose Sensors Fabricated by Electrochemical Polymerization of Phenols on Carbon Paste Electrodes Containing Ferrocene as an Electron Transfer Mediator. Anal. Sc.

[17] Nakabayashi Y., Yoshikawa H. (2000). Amperometric biosensors for sensing of hydrogen peroxide based on electron transfer between horseradish peroxidase and ferrocene as a mediator. Anal. Sc.

[18] Miscoria S.A., Barrera G.D., Rivas G.A. (2006). Glucose biosensors based on the immobilisation of glucose oxidase and polytyramine on rodhinised glassy carbon and screen printed electrodes. Sens. Actuators.

[19] Spatura T., Marcu M., Banu A., Roman E., Spataru N. (2009). Electrodeposition of platinum on polytyramine-modified electrodes for electrocatalytic applications. Electrochim. Acta.

[20] Adeloju S.B., Moline A.N. (2001). Fabrication of ultra-thin polypyrrole–glucose oxidase film from supporting electrolyte-free monomer solution for potentiometric biosensing of glucose. Biosens. Bioelectronics.

[21] Hall E.A.H., Gooding J.J., Hall C.E. (1995). Redox enzyme linked electrochemical sensors: Theory meets practice. Mikrochim. Acta.

[22] Trojanowicz M., Hitchman M.L. (1996). A potentiometric polypyrrole-based glucose biosensor. Electroanalysis.

[23] Guerrieri A., De Benedetto G.G., Palmisano F., Zambonin P.G. (1998). Electrosynthesized non-conducting polymers as permselective membranes in amperometric enzyme electrodes: a glucose biosensor based on a co-crosslinked glucose oxidase/overoxidized polypyrrole bilayer. Biosens. Bioelectronics.

[24] Adeloju S.B., Shaw S.J., Wallace G.G. (1996). Polypyrrole-based amperometric flow injection biosensor for urea. Anal. Chim. Acta.

[25] Adeloju S.B., Shaw S.J., Wallace G.G. (1997). Pulsed-amperometric detection of urea in blood samples on a conducting polypyrrole-urease biosensor. Anal. Chim. Acta.

[26] Gorchkov D.V., Soldatkin A.P., Maupas H., Martelet N., Jaffrezic-Renault C. (1996). Correlation between the electrical charge properties of polymeric membranes and the characteristics of ion field effect transistors or penicillinase based enzymatic field effect transistors. Anal. Chim. Acta.

[27] Nishizawa M., Matsue T., Uchida I. (1992). Penicillin sensor based on a microarray electrode coated with pH-responsive polypyrrole. Anal. Chem.

[28] Sohail M., Adeloju S.B. (2008). Electroimmobilization of nitrate reductase and nicotinamide adenine dinucleotide into polypyrrole films for potentiometric detection of nitrate. Sens. Actuators. B.

[29] Kulp T.J., Camins J., Angel S.M., Munkholm C., Walt D.R. (1987). Polymer immobilized enzyme optrodes for the detection of penicillin. Anal. Chem.

[30] Chao H.-P., Lee W.-C. (2000). A bioelectrode for penicillin detection based on gluten-membrane-entrapped microbial cells. Biotech. Appl. Biochem.

[31] Sharma S., Johnson R.W., Desai T.A. (2004). XPS and AFM analysis of antifouling PEG interfaces for microfabricated silicon biosensors. Biosens. Bioelectronics.

[32] Adeloju S.B., Shaw S.J., Wallace G.G. (1993). Polypyrrole-based potentiometric biosensor for urea part 1. Incorporation of urease. Anal. Chim. Acta.

[33] Grime J.K., Tan B. (1979). Direct titrations of antibiotics with iodate solution, part 1. Titration of some selected penicillins. Anal. Chim. Acta.

[34] Situmorang M., Gooding J.J., Hibbert D.B., Barnett D. (2001). Development of potentiometric biosensors using electrodeposited polytyramine as the enzyme immobilization matrix. Electroanalysis.

[35] Parag S.S., Shrikant A.S., Rekha S.S. (2008). Clavulanic acid: A review. Biotech. Advan.

[36] Grunwald L., Petz M. (2003). Food processing effects on residues: penicillins in milk and yoghurt. Anal. Chim. Acta.

[37] Cacciatore G., Petz M., Rachid S., Hakenbeck R., Bergwerff A.A. (2004). Development of an optical biosensor assay for detection of β-lactam antibiotics in milk using the penicillin-binding protein 2x*. Anal. Chim. Acta.

[38] Poghossian A., Abouzar M.H., Razavi A., Backer M., Bijnens N., Williams O.A., Haenen K., Moritz W., Wagner P., Schoning M.J. (2009). Nanocystalline-diamond thin films with high pH and penicillin sensitivity prepared on a capacitive Si-SiO_2_ structure. Electrochim. Acta.

[39] Siqueira J.R., Abouzar M.H., Poghossian A., Zucollotto V., Oliveira O.N., Schoning M.J. (2009). Penicillin biosensor based on a capacitive field-effect structure functionalized with a dendrimer/carbon nanotube multilayer. Biosens. Bioelectronics.

[40] Gaudin V., Fontaine J., Maris P. (2001). Screening of penicillin residues in milk by a surface Plasmon resonance-based biosensor assay: comparison of chemical and enzymatic sample pretreatment. Anal. Chim. Acta.

[41] Stred’ansky M., Pizzariello A., Stred’anska S., Miertus S. (2000). Amperometric pH-sensing biosensors for urea, penicillin, and oxalacetate. Anal. Chim. Acta.

